# Neuropilin-1 Protein May Serve as a Receptor for SARS-CoV-2 Infection: Evidence from Molecular Dynamics Simulations

**DOI:** 10.1021/acs.jpcb.4c03119

**Published:** 2024-07-16

**Authors:** Hoang Linh Nguyen, Ho Khac Hieu, Thai Quoc Nguyen, Nguyen Thi Ai Nhung, Mai Suan Li

**Affiliations:** † Institute of Fundamental and Applied Sciences, Duy Tan University, Ho Chi Minh City 700000, Vietnam; ‡ Faculty of Environmental and Natural Sciences, Duy Tan University, 03 Quang Trung, Hai Chau, Da Nang 550000, Viet Nam; § Institute of Research and Development, Duy Tan University, 03 Quang Trung, Hai Chau, Da Nang 550000, Viet Nam; ∥ Dong Thap University, 783 Pham Huu Lau Street, Ward 6, Cao Lanh City, Dong Thap 81000, Vietnam; ⊥ Department of Chemistry, University of Sciences, Hue University, Hue 530000, Vietnam; # Institute of Physics, Polish Academy of Sciences, al. Lotnikow 32/46, Warsaw 02-668, Poland

## Abstract

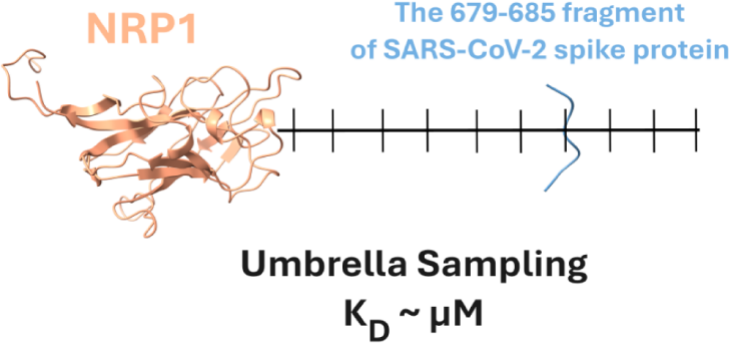

The binding of the virus to host cells is the first step in viral infection. Human cell angiotensin converting enzyme 2 (ACE2) is the most popular receptor for severe acute respiratory syndrome coronavirus 2 (SARS-CoV-2), while other receptors have recently been observed in experiments. Neuropilin-1 protein (NRP1) is one of them, but the mechanism of its binding to the wild type (WT) and different variants of the virus remain unclear at the atomic level. In this work, all-atom umbrella sampling simulations were performed to clarify the binding mechanism of NRP1 to the spike protein fragments 679–685 of the WT, Delta, and Omicron BA.1 variants. We found that the Delta variant binds most strongly to NRP1, while the affinity for Omicron BA.1 slightly decreases for NRP1 compared to that of WT, and the van der Waals interaction plays a key role in stabilizing the studied complexes. The change in the protonation state of the His amino acid results in different binding free energies between variants. Consistent with the experiment, decreasing the pH was shown to increase the binding affinity of the virus to NRP1. Our results indicate that Delta and Omicron mutations not only affect fusogenicity but also affect NRP1 binding. In addition, we argue that viral evolution does not further improve NRP1 binding affinity which remains in the μM range but may increase immune evasion.

## 1 Introduction

The outbreak of severe acute respiratory syndrome coronavirus 2 (SARS-CoV-2) has caused one of the devastating pandemics named COVID-19 by WHO.^1^ As of October 2023, COVID-19 has claimed more than 6.9 million lives out of approximately 771 million confirmed cases (https://covid19.who.int). Although existing vaccines and antibodies effectively control the pandemic, the mechanisms of viral infectivity and evolution at the atomic level are little known.^2^ A better understanding of these mechanisms will help us better prepare for a possible future pandemic. SARS-CoV-2 uses angiotensin-converting enzyme 2 (ACE2)^3,4^ as its primary receptor for host cell entry. Recently, CD147,^5^ KREMEN, ASGR1, CD209, CD209L, AXL, and neuropilin-1 (NRP1)^6−9^ were reported to be potential coreceptors of SARS-CoV-2. However, the molecular binding mechanism and the role of mutations of such variants as Delta and Omicron in their binding to coreceptors have not been studied.

The SARS-CoV-2 spike protein contains a fragment ^682^RRAR^685^, which is not present in its close relative SARS-CoV-1 that was responsible for the epidemic in 2002–2003. The experiment establishes that this fragment is a furin cleavage site although it is a suboptimal site since the classical furin cleavage site has the RRXRR motif (R is arginine and X is any amino acid), whereas the spike protein has a ^682^RRAR^685^ fragment.^10^ Furin cleavage at R685 splits the spike protein into 2 subunits S1 and S2, and the ^682^RRAR^685^ sequence belongs to the C-end rule (CendR) motif. This cleavage by furin triggers the formation of an open conformation of the spike protein to bind the ACE2 receptor^11^ and promotes cellular entry as well as pathogenesis of the SARS-CoV-2 virus.^10,12−14^ Moreover, ^682^RRAR^685^ is also a cleavage site of the TMPRSS2 protease.^15−17^ The structure of the SARS-CoV-2 spike protein indicates that the S1–S2 junction is exposed to the solvent.^11,18^ Therefore, proteolytic cleavage by furin or TMPRSS2 enhances solvent exposure of the C-terminus of the S1 subunit, facilitating the interaction of the C-terminus of the S1 subunit and the coreceptor as NRP1.

**Figure 1:**
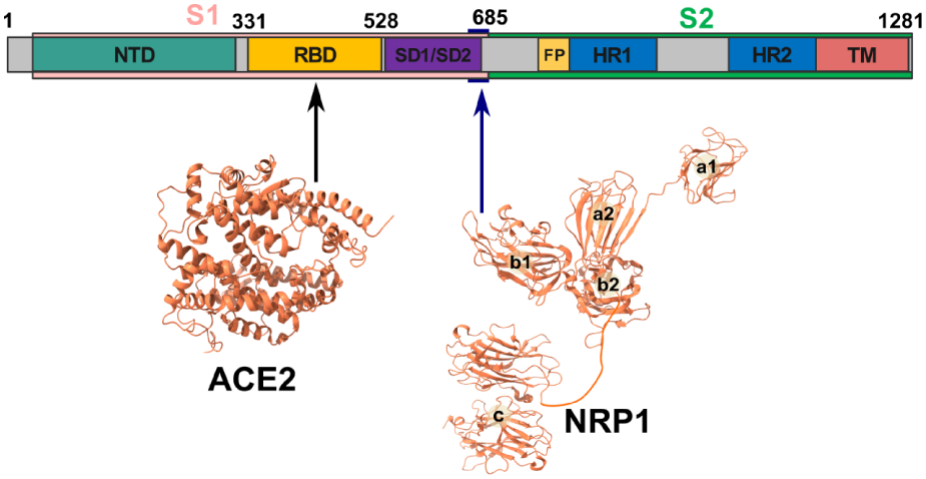
(Upper part) Spike protein sequence and its functional parts. (Lower part) Structure of ACE2 and NRP1. The arrows point to spike protein binding regions of ACE2 and NRP1.

Peptides with the CendR motif R/KXXR/K bind to the cell surface protein NRP1, as shown experimentally.^19^ After furin cleaves the spike protein to form the S1 and S2 subunit, the CendR motif ^682^RRAR^685^ of S1 binds directly to NRP1 in the b1 region (Figure 1).^6,7^ In addition, blocking the interaction of the C-terminal fragment of S1 with NRP1 by a small molecule inhibitor or monoclonal antibodies reduces the efficiency of virus infection.^7^ When NRP1 is coexpressed with ACE2, TMPRSS2 enhances significantly the viral infection.^6^ The binding of NRP1 b1 to the C-terminal fragment of S1 with residues 679–685 has a dissociation constant *K*_D_ of 20.3 and 13.5 μM at pH 7.5 and pH 5.5, respectively. These observations suggest that understanding the mechanism of the interaction between NRP1 and the S1 CendR motif ^682^RRAR^685^ plays an important role in unraveling the SARS-CoV-2 infection process. Transmission of SARS-CoV-2 is facilitated by viral RNA mutations, resulting in variants that differ in virulence and pathogenicity from the wild-type (WT) variant. The Delta variant emerged in October 2020 and caused a devastating surge in the pandemic.^20^ This variant contains a mutation at position P681 of the spike protein, which is in the region that binds to NRP1. Experiments have shown that the P681R mutation in the Delta variant enhances the cleavage of the spike protein, improving the fusogenicity performance of the virus.^21−23^ However, the effect of this mutation on NRP1 binding is still unknown. In November 2021, a novel variant of concern (VOC) called Omicron dominated over Delta in new COVID-19 cases. Omicron spike protein bears a large number of mutations in its spike protein, which enhances infectivity and immune evasion.^2,24^

There are many sublineages of Omicron,^2^ but in this work we focus on Omicron BA.1. It also has a mutation at P681 as Delta, but the His amino acid is replaced with Pro681. From now on, Omicron BA.1 will be called the Omicron. This variant has another N679K mutation in the spike protein. The P681H and N679K mutations do not improve fusogenicity and furin cleavage efficiency compared with WT and Delta.^23,25^ Moreover, Omicron inefficiently uses the TMPRSS2 protease to cleave the spike protein at 685 separating it into S1 and S2 subunits.^26^ These results suggest that mutations at the S1 CendR motif may play an important role in fusogenicity and the interaction of the viral spike protein with host cells.

In this work, we performed all-atom umbrella sampling simulations to investigate the binding of NRP1 and the spike protein fragments 679–685 of SARS-CoV-2 WT, Delta, and Omicron variants. The results show that Delta binds to NRP1 more strongly than do WT and Omicron. In accordance with the experiment,^8^ at pH = 5.5, the spike protein fragments 679–685 bind to NRP1 more tightly than at pH = 7.5. The van der Waals (vdW) interactions control the stability of NRP1 and the spike protein complex in all of the studied systems. The change in protonation state due to pH change alters the electrostatic interaction of NRP1 and the spike protein fragments 679–685, resulting in different binding affinities of NRP1 to different targets. Since other Omicron lines, such as BA.2, BA.3, BA.4, and BA.5, have the same mutations in fragments 679–685 as BA.1, our result can be applied to them. The fragments 679–685 of the S1 subunit of the spike protein are also called the S1 CendR motif.

## 2 Materials and Methods

### 2.1 Initial Structures

**Figure 2:**
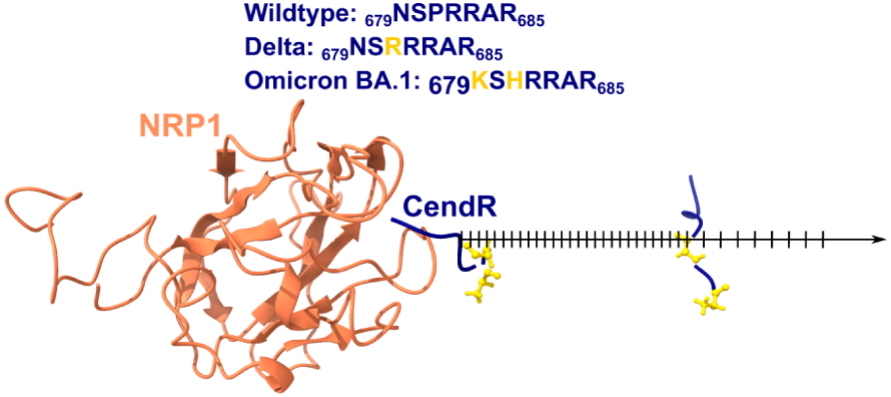
(Upper part) Amino acid sequence of S1 CendR motif, the mutations are in gold. (Lower part) Initial structure for simulations, the black arrow represents the pulling direction. The S1 CendR motif structures represent the configuration at the beginning and at the time point when the window interval is changed to 0.1 nm. Two mutated positions are shown in gold balls and sticks.

The structure of the NRP1 b1 domain in complex with fragments 679–685 of the WT spike protein of SARS-CoV-2 was obtained from the Protein Data Bank (PDB) under code 7JJC.^8^ In this work, we set up 3 such complexes for the WT, Delta, and Omicron variants. From the 7JJC structure, the P681R mutation in the spike protein was created for Delta, while the N679K and P681H mutations were created for the Omicron. The GROMACS 2022 package was used to perform molecular dynamics (MD) simulations.^27^ The systems were solved with TIP3P water molecules in a rectangular box with a size of 8 × 8 × 20 nm.^28^ To neutralize the system, Na^+^ and Cl^–^ counterions were added at a concentration of 0.15 M. The AMBER14SB force field was used to parametrize proteins.^29^ A representation of the initial structure is shown in Figure 2. The protonation states of the residues are determined by the PROPKA3 program.^30^

### 2.2 Steered Molecular Dynamics Simulations

We first performed steered molecular dynamics (SMD) to obtain conformations along the reaction coordinate, and these conformations were used as starting structures for umbrella sampling simulations. The reaction coordinate is defined as the distance between the centers of mass of the S1 motif, CendR, and NRP1. In SMD, the S1 CendR motif is pulled out from the NRP1 binding region along the reaction coordinate (note that since the S1 CendR motif binds to the surface of NRP1, the direction of the pull is straight or can be found using our zigzag algorithm in just one step.^31^ For simplicity, the system was rotated to align the reaction coordinate with the *z*-axis of the Cartesian coordinate system.

An external force is applied to a dummy atom connected to the atom closest to the center of mass of the S1 CendR motif by a harmonic spring. The spring constant was chosen to be 600 kJ/mol/nm^2^, which is a typical value for AFM experiments.^32^ The pulling speed was set to 0.5 nm/ns.

The complexes were energy minimized using the steepest descent algorithm followed by equilibration sequentially in the NVT and NPT ensembles for 1 and 5 ns MD simulations, respectively. The temperature was kept at 300 K by the v-rescale algorithm and pressure was maintained at 1 atm by the c-rescale algorithm.^33,34^ Five independent SMD trajectories were carried out, and the snapshots collected in the run with the rupture force closest to the average rupture force were selected as starting structures for umbrella sampling.

### 2.3 Umbrella Sampling Simulations

The displacement windows for the US simulation are shown in Figure 2. The first 2 nm of the reaction coordinates along the pulling direction is divided into 40 windows of 0.05 nm each, and the last 1.5 nm is divided into 15 windows of 0.1 nm each. In total, we have 55 windows for each complex. This asymmetric arrangement was used because, in the first 2 nm of the displacement, where the rupture force occurs, the NRP1–CendR interaction is stronger than in the second part. To hold CendR around the center of each window, a harmonic potential with a force constant of 600 kJ (mol nm^2^)^−1^ was used. As in the SMD simulations, the temperature and pressure were maintained at 300 K and 1 atm, respectively. For each window, a traditional MD simulation of 150 ns duration was performed. The potential of mean force (PMF) was analyzed using the gmx wham tool of the GROMACS package.^35^ Errors were estimated using the bootstrap method in gmx wham.

## 3 Results and Discussion

### 3.1 S1 CendR Motif of Delta Variant Binds NRP1 More Strongly Than WT and Omicron Variant

Figure S1A shows the time dependence of the root-mean-square displacement (RMSD) of all atoms relative to the initial structure of the complex of NRP1 and WT CendR. The result obtained from the umbrella sampling simulation is shown for one selected window because similar behavior is true for other windows. At pH 7.5, the complex reaches equilibrium in approximately 50 ns. At pH 5.5 the fluctuations are smaller and equilibrium is reached earlier, but for comparison we used snapshots taken after 50 ns to calculate the PMF in both cases. In order to clearly demonstrate the convergence of umbrella sampling simulations we have considered the WT case in more detail. The WT PMF was calculated at pH 5.5 and 7.5 in the time interval [50–100 ns] and compared with the result obtained in the time interval [50–150 ns] (Figure S1B). Since the PMF profiles in both time windows are almost identical, the umbrella sampling simulation converges, and we now present only the results obtained in a wider time window. It can be shown that this procedure is also valid for the Delta and Omicron variants.

**Figure 3:**
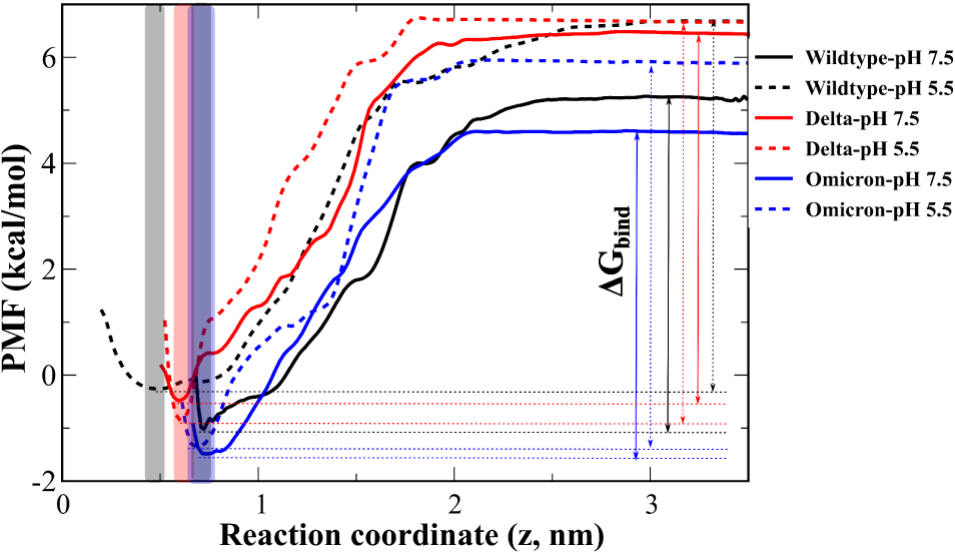
Potential of mean force (PMF) from umbrella sampling simulations for WT, Delta, and Omicron at pH 5.5 (dashed lines) and pH 7.5 (solid lines). The binding energy Δ*G*_bind_ is defined as the barrier between the bound state and transition state, which is equal to the difference between the minimum and maximum PMF values. The snapshots in translucent colored areas around the minimum are used to analyze interaction between fragments of spike protein and NRP1. Snapshots collected in translucent colored areas around the minimum are used to analyze the interaction between the spike protein and NRP1.

PMF profiles obtained for three complexes at pH 5.5 and 7.5 in the [50–150 ns] time window are shown in Figure 3. The binding energy Δ*G*_bind_ is defined as the barrier between the bound state and the transition state, which is equal to the difference between the minimum and maximum PMF values. Experimental Δ*G*_bind_ was extracted from *K*_D_ using the formula Δ*G*_bind_ = *k*_B_*T* ln(*K*_D_), where *K*_D_ is measured in mol and *T* = 300 K. For convenience, the *K*_D_ values obtained from the experiment and our simulation are also shown in Table S1.

The binding free energies of the S1 CendR motif to NRP1 at pH 7.5 and pH 5.5, which were obtained from experiment^8^ and umbrella sampling simulations for WT, Delta, and Omicron, are shown in Table 1. Both experiment and simulation show that in the case of WT, the binding affinity at pH 5.5 is slightly higher than that at pH 7.5. This trend also holds for Delta and Omicron, but the difference in Δ*G*_bind_ at different pH is more pronounced for Omicron, which is likely due to Omicron having more mutations in the S1 CendR motif. Overall, Delta binds to NRP1 more strongly than does WT and Omicron (Table 1).

**Table 1:** Binding Free Energies (kcal/mol) Obtained from Experiment and Umbrella Sampling Simulations^a^

	pH 5.5			pH 7.5		
system	wildtype	Delta	Omicron BA.1	wildtype	Delta	Omicron BA.1
experiment^8^	–6.63			–6.41		
simulations in this work	–6.92 ± 0.41	–7.52 ± 0.83	–7.18 ± 0.51	–6.24 ± 0.32	–6.85 ± 0.34	-6.01 ± 0.28

a In the experimental case Δ*G*_bind_ was extracted from *K*_D_ using the formula Δ*G*_bind_ = *k*_B_*T* ln(*K*_D_), where *K*_D_ is measured in mol and *T* = 300 K. The errors are obtained from bootstrap analysis in the gmx wham tool.

### 3.2 van der Waals Interaction Plays an Important Role in the Complex of NRP1 and S1 CendR Motif

To investigate the binding mechanism of the S1 CendR motif to NRP1, we analyzed interactions around the minimum of the PMF curve (translucent colored areas in Figure 3). Using the MM-PBSA method,^36^ the interaction energy between NRP1 and CendR, including electrostatic, vdW, and polar solvation components, was calculated and shown in Table 2.

**Table 2:** Interaction Energy between Fragments of Spike Protein and NRP1 Obtained from Snapshots in Transparent Regions in Figure 3^a^

	pH 5.5			pH 7.5		
interaction (kcal/mol)	wildtype	Delta	Omicron	wildtype	Delta	Omicron
electrostatic	–24.91 ± 6.24	16.86 ± 8.27	166.58 ± 6.41	–241.23 ± 5.97	–250.92 ± 3.44	-212.16 ± 2.01
van der Waals	–35.83 ± 0.19	–33.84 ± 4.04	–24.66 ± 2.92	–18.13 ± 0.41	–26.42 ± 0.18	-24.07 ± 0.35
polar solvation	39.97 ± 2.10	–12.68 ± 9.05	–166.90 ± 6.04	240.80 ± 15.67	249.58 ± 9.12	213.98 ± 12.89
total	–20.77 ± 6.78	–29.66 ± 7.71	–24.98 ± 6.55	–18.56 ± 6.05	–27.76 ± 6.27	-22.25 ± 6.55

a The errors represent standard deviations.

At pH 7.5, the electrostatic and polar solvation energies are greater than the vdW energy. However, these interactions balance each other, which leads to the dominance of vdW forces. The Delta variant has the strongest vdW interaction, resulting in the lowest total energy. At pH 5.5, the electrostatic and polar solvation terms are smaller than their counterparts at pH 7.5. This is due to a change in the protonation state of His residues in the NRP1 and Omicron S1 CendR motif when the pH decreases from 7.5 to 5.5, which increases the number of positively charged residues. At pH 7.5, NRP1 has zero charge, while at pH 5.5 NRP1 has a +6e charge due to the protonated His amino acid.

At pH 7.5, the S1 CendR motif of WT, Delta, and Omicron has a charge of +3e, +4e, +4e, respectively, while at pH 5.5 these values are +3e, +4e, +5e. This effect demolishes the attractive electrostatic interaction between the S1 CendR motif and NRP1 at pH 5.5 compared to pH 7.5, but makes the complex more readily soluble at pH 5.5. As a result, at pH 5.5 only WT has negative electrostatic energy and Omicron has large repulsive electrostatic interaction (Table 2).

The Delta variant has a greater balance between repulsive electrostatic interaction and polar solvation energy compared to the WT and the Omicron at pH 5.5. Therefore, protonation induced by the pH variation leads to changes in electrostatic and polar solvation terms. The vdW still dominates the total energy with a lower value in all systems at pH 5.5 than in their counterparts at pH 7.5. However, in the case of WT, the sum of the electrostatic and polar solvation terms is reduced to a greater extent than in the other variants, causing its total energy to be higher than Omicron and Delta. Therefore, the vdW interaction rules the stability of the CendR–NRP1 complex, but the electrostatic interaction determines the difference between the variants.

### 3.3 Important Residues of S1 CendR Motif in Interaction with NRP1

**Figure 4:**
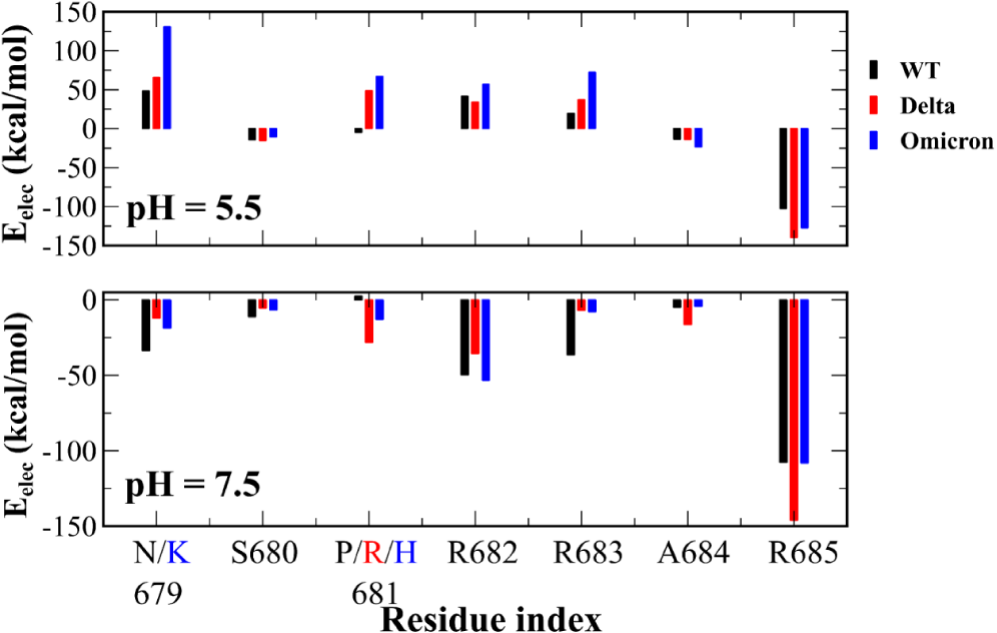
Electrostatic interaction energy *E*_elec_ (kcal/mol) per residue of the S1 CendR motif. The result was obtained using snapshots collected in the translucent colored regions of Figure 3.

**Figure 5:**
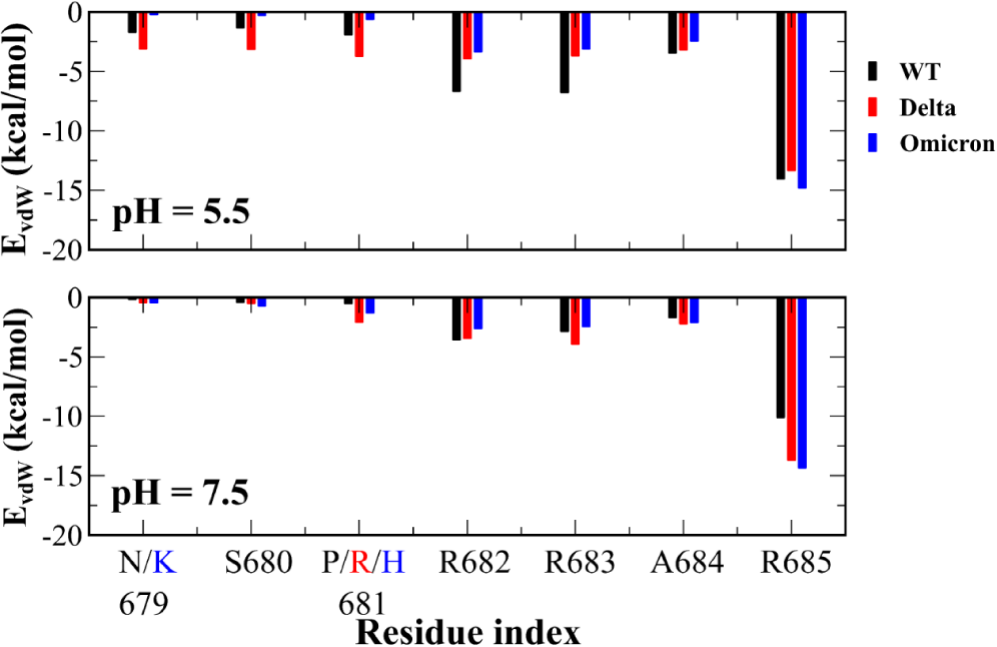
vdW interaction energy *E*_vdW_ (kcal/mol) per residue of the S1 CendR motif. The result was obtained using snapshots collected in the translucent colored regions of Figure 3.

The nonbonded interaction energy of residues of the S1 CendR motif was assessed in the translucent colored areas in Figure 3. At pH 7.5, all CendR residues of all variants have the negative electrostatic interaction (Figure 4), i.e., they stabilize the complex. For WT, residues N679, R682, R683, and R685 have strong electrostatic interaction at this pH, with residue R685 dominating. At pH 5.5, residues N679, R682, and R683 have repulsive electrostatic interaction, making the complex less stable. Residue R685 still makes the largest contribution to the attractive electrostatic interaction, since S680, R681, and A684 have negligible electrostatic energy. Changing the pH does not qualitatively change the per-residue contribution of vdW interactions (Figure 5). R685 also dominates in the vdW interaction at both pH values. R682, R683, and A684 make a significant contribution to the vdW energy, but to a lesser extent than R685.

In the case of Delta at pH 5.5, the P681R mutation significantly increases the electrostatic interaction from negative to approximately +50 kcal/mol (Figure 4). The electrostatic interaction between R685 and NRP1 is enhanced in Delta, while for other residues, it changes only slightly. At pH 7.5, residues R681, A684, and R685 of Delta improve electrostatic interaction compared to WT, while for N679, S680, R682, and R683 this interaction is weakened. This result suggests that the P681R mutation affects the configuration of neighboring residues, leading to a decrease in the level of electrostatic interactions. At pH 5.5, residues 679–681 of Delta enhance the vdW interaction, whereas the remaining residues reduce it (Figure 5). At pH 7.5, Delta enhanced the interaction of vdW residues 681, 683–685 with NRP1, but little effect was observed for other residues.

In the case of Omicron, the N679K mutation dramatically increases the electrostatic repulsive energy at pH 5.5 (Figure 4). The P681H mutation makes the electrostatic interaction energy change from negative to about +75 kcal/mol, which is a stronger effect than P681R of Delta at pH 5.5. Residues R682, R683, and A684 of the Omicron variant have higher electrostatic energy values than their WT counterparts. At pH 7.5, the N679K mutation weakens the attractive electrostatic interaction and P681H increases the interaction energy. Other residues of Omicron’s S1 CendR motif of Omicron reduce electrostatic energy (680, 683, 684) or have little effect on electrostatic interactions (682, 685). The vdW interaction of Omicron residues is weaker than that of WT and Delta, with the exception of R685 at pH 5.5. Omicron’s R685 residue of Omicron has a stronger vdW interaction with NRP1 than WT and Delta at both pH values. Other residues at pH 7.5 have similar vdW energies compared to those of WT and Delta.

Thus, residue R685 plays an important role in the stability of the NRP1–CendR complex. The Delta and Omicron mutations primarily affect electrostatic interactions. At pH 5.5, mutations in these variants weaken the electrostatic interaction, while at pH 7.5, N679K reduces the attractive electrostatic interaction, and P681H and P681R increase it.

### 3.4 The Number of Hydrogen Bonds Decreases with Increasing pH

**Figure 6:**
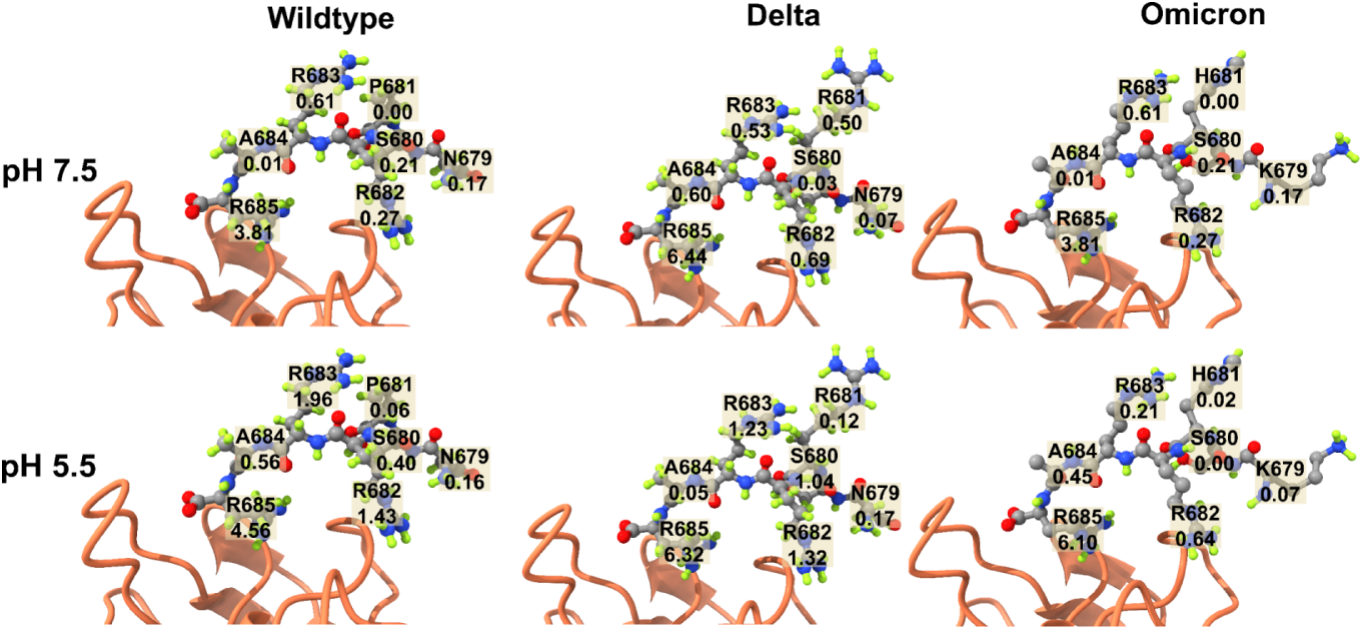
Average number of hydrogen bonds of S1 CendR motif residues. The residues of this motif are represented by balls and sticks while NRP1 is represented by cartoon.

The total number of hydrogen bonds (HB) between NRP1 and the S1 CendR motif of the three variants at pH 5.5 is greater than at pH 7.5 (Table 3). The Delta variant produces more HB than the others at both pH values, while Omicron does not increase HB compared to WT. These results are consistent with umbrella sampling results that the S1 CendR motif of the Delta variant binds more tightly to NRP1 than WT and Omicron. The average number of HB per residue of the S1 CendR motifs at pH 5.5 is higher than that at pH 7.5 (Figure 6), confirming the higher binding affinity (lower binding free energy) at lower pH. In the WT case, HBs are formed mainly at residues R682, R683, and R685 under both pH conditions. At pH 5.5, WT A684 and S680 residues form more HB than at pH 7.5, while WT residues N679 and P681 have little change in HB.

**Table 3:** Average Total Number of Hydrogen bonds between the S1 CendR Motif and NRP1^a^

	pH 5.5			pH 7.5		
	wildtype	Delta	Omicron	wildtype	Delta	Omicron
number of HBs	9.13	10.25	7.49	5.08	8.86	5.08

a The results were obtained from snapshots collected in transparent regions in Figure 3.

In the case of the Delta variant, the amount of HB is higher than that of WT at pH 5.5 and 7.5, which is consistent with the binding free energy results. The P681R mutation increases the amount of HB compared to WT at pH 7.5, but at pH 5.5, this effect is negligible. As with the WT, residues R682, R683, and R685 have high HB populations at both pH values. At pH 7.5, residue A684 forms more HB than at pH 5.5, which contrasts with WT. Residues N679, S680, R682, and R683 form more HB at pH 5.5 than at pH 7.5. Residue R685 in Delta improves HB formation compared to that in WT at both pH values.

The P681H and N679K mutations in the Omicron variant have little effect on the HB between the S1 motif of CendR and NRP1 (Figure 6). This may resemble the fusogenicity of Omicron, which is weaker than WT.^23^ The P681R mutation makes a more pronounced contribution to HB formation than the P681H and N679K mutations. The effect of improving P681R and reducing the effect of mutations P681H and N679K on the cleavage TMPRSS2 and fusogenicity were observed experimentally.^23,26,37,38^ Here, we found a similar effect of these mutations on the NRP1 binding of Delta and Omicron variants.

## 4 Conclusion

Using umbrella sampling with all-atom models, we obtained the binding free energy of the S1 CendR motif of WT, Delta, and Omicron interacting with NRP1. Our result obtained for WT is consistent with the experiment showing that increasing pH slightly decreases binding affinity, and this pH dependence also holds for Delta and Omicron. The Delta variant binds to NRP1 more strongly than WT and the Omicron at both pH values, which have the same binding affinity. The vdW interaction controls the stability of the complexes, but the electrostatic interaction causes differences between the binding free energies of the variants.

R685 residue plays an important role in the NRP1–CendR interaction at both pH values. At pH 5.5, mutations N679K and P681H of the Omicron and P681R of the Delta weaken the electrostatic interaction. At pH 7.5, N679K reduces attractive electrostatic interaction, and P681H and P681R increase it. CendR residues of Delta form more HBs with NRP1 than WT and Omicron. At pH 5.5, the amount of HBs increases in all systems, which is consistent with the result obtained for binding free energy from umbrella sampling simulations. These results suggest that the Delta variant not only enhances the fusogenicity but also improves NRP1 binding compared with those of WT and Omicron. Our result for the Omicron BA.1 lineage can be applied to other lineages with the same mutation at the S1 CendR motif, such as BA.2, BA.3, BA.4, and BA.5.

Both experiment^8^ and our simulations indicate that the binding affinity of the SARS-CoV-2 spike protein interacting with NRP1 can be characterized by the dissociation constant *K*_D_ in the μM range (Table S1). On the other hand, the interaction of the spike protein with human ACE2 has a typical *K*_D_ value ∼nM,^2,18,39^ suggesting that the spike protein binds to NRP1 less strongly than to ACE2. From a biophysical point of view, this fact may indicate that NRP1 is less effective in viral infection compared to ACE2. However, NRP1 may become important when the level of ACE2 expression is low.

Table S2 shows mutations in the S1 CendR motif of the different variants. Many lineages, such as Beta and Gamma mutations, do not occur in this region and are not shown. Since the maximum number of mutations is 2, we expect that the binding affinity for NRP1 will not change significantly during evolution and *K*_D_ will remain in the μM range, as in the cases studied in this work. This is consistent with our recent observation^40^ that evolution does not improve the binding affinity of SARS-CoV-2 to human ACE2, but may increase immune evasion.

## Supporting Information Available

Figure S1: time dependence of the root-mean-square displacement (RMSD) of all atoms relative to the initial structure of the WT complex; Table S1: *K*_D_ (μM) of spike protein and NRP1 complex from experiment and simulation; Table S2: mutations in the CendR motif of the S1 subunit of various SARS-CoV-2 variants (PDF)
