# Supplementary material for: Neuropilin-1 Protein May Serve as a Receptor for SARS-CoV-2 Infection: Evidence from Molecular Dynamics Simulations
Source: J Phys Chem B. 2024 Jul 16;128(29):7141–7. doi: 10.1021/acs.jpcb.4c03119 (PMC11284781; doi:10.1021/acs.jpcb.4c03119)
Supplement: Supplementary file 1 — jp4c03119_si_001.pdf [file jp4c03119_si_001.pdf]

Neuropilin-1 Protein May Serve as a Receptor for SARS-CoV-2 Infection: Evidence from Molecular Dynamics Simulations

Hoang Linh Nguyen[1,2,*], Ho Khac Hieu[2,3], Thai Quoc Nguyen[4,*], Nguyen Thi Ai Nhung[5], and Mai Suan Li[6,*]

[1]Institute of Fundamental and Applied Sciences, Duy Tan University, Ho Chi Minh City 700000, Vietnam

[2]Faculty of Environmental and Natural Sciences, Duy Tan University, 03 Quang Trung, Hai Chau, Da Nang 550000, Viet Nam

[3]Institute of Research and Development, Duy Tan University, 03 Quang Trung, Hai Chau, Da Nang 550000, Viet Nam

[4]Dong Thap University, 783 Pham Huu Lau Street, Ward 6, Cao Lanh City, Dong Thap, Vietnam

[5]Department of Chemistry, University of Sciences, Hue University, Hue 530000, Vietnam

[6]Institute of Physics, Polish Academy of Sciences, al. Lotnikow 32/46, 02-668, Warsaw, Poland

*Email: nguyenhoanglinh9@duytan.edu.vn, nqthai@dthu.edu.vn, masli@ifpan.edu.pl

SUPPORTING INFORMATION

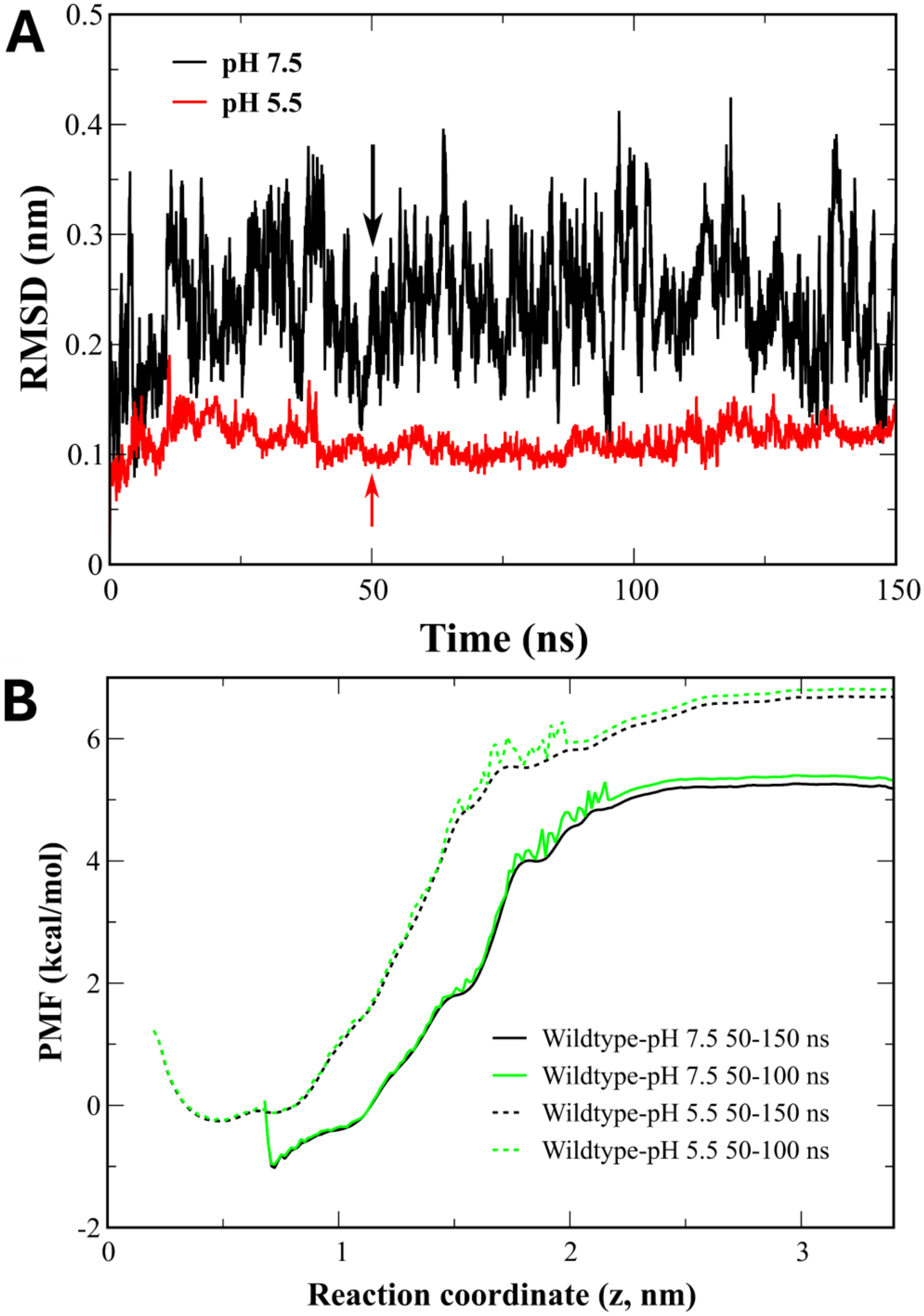

Figure S1. (A) Time dependence of the root mean square displacement (RMSD) of all atoms relative to the initial structure of the WT complex. Results were obtained from umbrella sampling simulations for a selected window at pH 5.5 and 7.5. The arrow indicates the 50 ns time point when the system reached equilibrium. (B) PMF profiles obtained for WT at pH 5.5 and 7.5 in [50:100] and [50:150] ns time windows.

Table S1: K_D (μM) of spike protein and NRP1 complex from experiment and simulation.

System	pH 5.5			pH 7.5		
	Wildtype	Delta	Omicron BA.1	Wildtype	Delta	Omicron BA.1
Experiment [1]	13.0			20.3		
Simulations in this work	9.09 ± 6.25	3.32 ± 4.63	5.88 ± 5.03	28.44 ± 15.27	10.22 ± 5.83	41.84 ± 19.65

1. Daly, J.L.; Simonetti, B.; Klein, K.; Chen, K.-E.; Williamson, M.K.; Antón-Plágaro, C.; Shoemark, D.K.; Simón-Gracia, L.; Bauer, M.; Hollandi, R. Neuropilin-1 is a host factor for SARS-CoV-2 infection. *Science* **2020**, *370*, 861-865.

Table S2: Mutations in the CendR motif of the S1 subunit of various SARS-CoV-2 variants. The latest variant JN.1 belongs to the BA.2.86 sublineage.

Variant	Mutations
Alpha	P681H
Delta	P681R
Omicron BA.1, BA.2, BA.3, BA.4, BA.5, XBB	N679K, P681H
Omicron BA.2.86, JN.1	N679K, P681R
